# Removal of the Parotid Gland, by Professor Pancoast

**Published:** 1847-07

**Authors:** Ellerslie Wallace

**Affiliations:** Demonstrator of Anatomy in Jefferson Medical College; 309 Walnut Street, Philadelphia


### MEDICAL EXAMINER,

AND

### RECORD OF MEDICAL SCIENCE.

NEW SERIES.—No. XXXI.—JULY, 1847.

### ORIGINAL COMMUNICATIONS.

*Removal of the Parotid Gland, by* PROFESSOR PANCOAST. Reported by ELLERSLIE WALLACE, M. D., Demonstrator of Anatomy in Jefferson Medical College.

To the Editor of the Medical Examiner.

*Dear Sir*,—I send you an account of an operation for the extirpation of the parotid gland, performed by Dr. Pancoast, on Saturday June 5th, in the presence of Dr. Sharpless of Downingtown, Pennsylvania, Dr. Dorr of Boston, and Drs. Togno, J. V. Patterson, of this city, and myself, besides Messrs. Banks, Prince, Horner, and several others, students of medicine.

The patient was a Mrs. Twining, from Newport, Bucks county, Pennsylvania, a woman of 60 years of age. According to her statement and that of her friends, the disease commenced upwards of ten years ago as a swelling of the gland, of an acute character, simulating ordinary parotitis. After the acute symptoms had passed away, the gland did not return to its normal size, but remained a little enlarged for a few years. It then began to increase in size, and she applied to Dr. Smith of Newport, who informed her that it was an enlargement of the parotid gland, for which he did not at the time advise any operation. Its growth increasing more rapidly within the last year, and its increase of growth being accompanied by much distress from severe shooting pains about the face and forehead, she came to Philadelphia to seek surgical aid; and, consulting Dr. Pancoast, gladly consented to an operation in hope of a cure.

The tumour was on the right side of the face, nodulated and irregular in its external aspect, and appearing about half the size of a man's fist. It extended from a little above the zygoma, to a short space below the angle of the jaw—passing forward over the greater part of the masseter muscle, and backward under the ear, so as to elevate and press posteriorly the anterior border of the ear; it likewise nearly surrounded the auditory meatus, and also overlapped the insertion of the sterno-cleido-mastoid. When grasped firmly, it was found but slightly moveable, deeply fixed, and firm in its texture, except at its upper part, where there seemed a local point of softening.

None of the surrounding lymphatic glands seemed at all involved. The complexion of the patient was somewhat straw colored, though she appeared vigorous for her age.

*Operation.*—The patient was placed on her left side, with the head and shoulders elevated, and her head well turned towards the left shoulder. The tumour was exposed by a single incision shaped somewhat like the Italic *ſ* reversed: it was commenced above the top of the ear, and carried forward and downward to near the centre of the tumour, then in a direction sloping slightly backwards to just below the lobe of the ear, when it was again directed forward, downward and nearly vertically, leaving a concavity in front, and terminating about an inch and a half below the base of the jaw, and somewhat within the inner edge of the sterno-mastoid. The dissection was then commenced by reverting the flaps so as to expose the tumour, and continued by separating the diseased mass first above, then posteriorly, next anteriorly, and lastly below. Some vessels bled from the surface of the tumour, as well as some small arterial branches from the flap, but by pressure of the fingers and the application of a few ligatures, all material hemorrhage was arrested.

Dr. Pancoast now sought for the external carotid artery, with a view of placing a ligature upon it, near its entrance into the tumour; this required a slight increase in length of the first incision, as from the size and attachments of the tumour, it was somewhat difficult to reach the vessel. It was isolated, however, with its vena comes, and the two were raised on the director, and a Physick's aneurismal needle armed with a ligature passed under them, along the groove in the director, and both secured in the loop. From this moment to near the conclusion of the operation, there was very trifling hemorrhage. The vessels were now cut beyond the ligature, and while strong traction was made upon the tumour, Dr. P. detached it from its connexions to a still greater distance below. The patient complained much of the pain caused by the upward traction. The tumour was next loosened to a greater extent above, as well as posteriorly and anteriorly.

The central part of the tumour, deeply seated, was the last part detached; and a strong jet of blood, by retrogression from the internal maxillary artery as the final cuts were made, required that a ligature should be applied to the divided vessel. This ligature, with two on smaller bleeding vessels, and the one on the carotid artery, were all that were left at the conclusion of the operation.

A small piece of diseased structure being discovered after the thorough cleansing of the wound, near the bottom of the cavity, it was removed by the handle and blade of the scalpel. As far as was possible, the handle of the scalpel was used during the operation, but for the most part the attachments were so firm as to require the cutting edge. The constant firm traction directed by Dr. Pancoast, was of much value in facilitating and in hastening the extirpation of the diseased mass.

The depth of the wound was very great, as well as its extent. It was six inches in length, exposing the greater part of the masseter muscle, a part of which, being adherent to, was removed with the tumor, and a small portion of the buccinator was also laid bare. The under surface of the internal pterygoid was exposed, as well as the entire ramus of the jaw posterior to the masseter muscle; the ligaments of the temporo-maxillary articulation were also laid bare, on their outer, lower and inner surface, and the condyle could be seen sliding forward in its socket when the mouth was opened. The finger being placed on the styloid process of the temporal bone, (which was exposed its whole length,) and carried downward, the contraction of the styloid muscles could be distinctly felt. A part of one of the styloid muscles, which was embraced by the tumour, was removed with it. The insertion of the sterno-cleido-mastoid into the mastoid process was also plainly shown. There was paralysis of the side of the face and of the orbicularis oculi, induced by the division of the portio dura—this nerve having been removed with the diseased structure. The lips of the wound were approximated by suture, and pressed down into the deep cavity by a compress of lint spread with cerate; another compress was laid over the entire length of the incision, and strips of adhesive plaster applied to keep the sides of the cavity in contact. The patient was a good deal exhausted at the close of the dressing, and took about ℥j. wine in some water ; reaction soon came on, and she pronounced herself comfortable.

Dr. Pancoast invited me to visit the case after the operation, and upon no occasion has there been any unpleasant symptom, either constitutional or local. Her appetite has been good, she has rested well, had no fever, nor local pain nor soreness enough to induce any complaint. We examined the wound on the fifth day after the operation, and the upper and lower part, for three-fourths of an inch, had united by first intention, and so favorable was its appearance, that the centre, where the first compress had been placed, was not disturbed. On the tenth day the first entire dressing was made, and on the twelfth, the second. There had been no discharge of matter, except a little that hardened on the ligatures, and there was scarcely any odour from the wound. Union by first intention has been complete—closely embracing the ligatures, the integuments being sunk down in the deep fossa left by the removal of the diseased gland.

Since the fifth day from the operation, the patient has dressed and set up daily. Very respectfully yours,

Ellerslie Wallace,

*June* 18, 1847. 309 Walnut Street, Philadelphia.

